# Obsessed with fertility

**DOI:** 10.1093/eurpub/ckae134

**Published:** 2024-08-27

**Authors:** Vasiliy Vlassov

**Affiliations:** Society for evidence based medicine, Moscow 101000, Russian Federation

Recent publications of the UN population projections as well as the Global Burden of Disease estimations [1] have not attracted the world’s attention. While the total world population will be stabilized, the total fertility rate (TFR) will decline below the replacement level in most countries. Many countries with negative natural population growth soften the problem by immigration. But it is not acceptable in countries with strong anti-immigration standing of the government and society, like Russia.

Russia not only has a very low population density—like Canada, it also has a huge territory and long borders with countries that are not very friendly. Therefore, the Russian government is working on increasing the life expectancy (LE) and fertility. When LE started to decline in 1964, the USSR classified the statistics of the neonatal mortality. Data were opened again after the collapse of the USSR, but not for long. In 2012, the “year of cardiovascular diseases” was announced, and in 1 year, the structure of mortality had changed. [2] Since 2014, the Human Mortality Database has not included data from Russia. Since 2024, Russian statistical agency classified detailed data on mortality from external causes. Worst of all, adult life expectancy has not increased much during the last 70 years.

To increase the birth rate, the government acted against abortions. Under communist rule, the abortion rate was very high, but after 1991, it declined to the level of Western European countries—thanks to better access to contraceptives. To reduce the abortion rate further, the government, lawmakers, and the Orthodox Church opted for suppression of access to medical abortions. Pregnant women seeking abortion were obliged to watch the ultrasound of the fetus, the execution of abortion to be delayed after the first visit, and women were obliged to visit a “psychologist.” The Ministry of Health advises psychologists to change the abortion decision of the woman. Also, in some regions of Russia, meeting with the Orthodox Church priest before abortion is compulsory. Now regional statistics report the numbers of women who changed their mind after advice to keep the pregnancy. The government limited the list of social indications for medical abortions, and limited access to the drugs used in the medicinal abortion. In 2023, parliament drafted the law declaring the fetus a live person with the right to have their life protected, and called the Ministry of Health to shorten the voluntary abortion window from 12 to 9 weeks.

The nudging to abort the pregnancy became forbidden. In the regional legislation wording like “safe abortion” or presenting a fetus as not a live person are examples of nudging. In public, press, and in the Parliament, there are calls to stop the provision of medical abortions in state clinics, or to bar private clinics from providing abortions. While the national law is yet to be changed, in some regions of Russia, private clinics “voluntarily” canceled their licenses for the provision of abortions. All this pressure did not change the continuous decline of the abortion rate. This activity was destined to fail from the beginning, but it was the line of the additional pressure on women.

Since 2023, the national program of “dispanserization” (periodical check-ups) was extended: The “evaluation of the reproductive health” was introduced. Additional screenings, like evaluation of the sperm quality, etc. — the whole range of interventions not based on scientific evidence.

During last 20 years, Government has employed different means to increase the birth rate—by the provision of monetary premiums, reduced rate credits for apartment purchase, and lots of “moral” stimulation. *In vitro* fertilization became freely available. The multi-child family is promoted as an example of good citizenship, as a symbol of belonging to the orthodox national style. Despite advice from demographers and public health specialists, government and propaganda outlets promote early pregnancy (before age 20), and promote the image of a good woman as one belonging to childbearing and family, but not involved in long education. The Minister of Health in July 2024 called the delay in birth of the first child a sin, and said that later first pregnancies lead to infertility and miscarriages. Voluntary child-free families pictured as belonging to the “movement” alien to Russian national values.

“Sexual deviations” became the major enemies of the state. First, pedophilia and “propaganda of homosexualism to minors” was criminalized and became the reason for the first censorship of the internet. In a short time, any “propaganda,” and any presence of LGBTQ symbols, including wearing earrings with a rainbow, became a crime. Recently, the Supreme Court declared the “international LGBT movement” an alien extremist organization. Transpersons were addressed as alien perverts. The number of medical facilities that can consult people in need of gender-affirming care was limited, and conversion was completely stopped. In August 2024, the draft new version of the medical contraindications for car driving was published. In this version, ICD codes F65.0–F65.8 and F66.0–F66.8 are detailed. Specialists from the psychiatric institute responsible for this explained that these codes are applicable only in case of chronic severe disorders—whatever it means concerning e.g. voyeurism. This may not be ignored as a funny bureaucratic *lapsus lingue*. These psychiatrists responsible for the forensic consultations are from the same Serbsky institute, where dissidents of the Soviet time were found schizophrenics.

The list of the strange excursions against LGBTQ and freedom of access to information is long. Recently, the Ministry of Health canceled the transition to ICD11 because the “sexual perversions” are “normalized” in it. Fiction books with LGBTQ chapters/lines are cleared from libraries and book shops. Old TV series are censored by cutting out the scenes where gay themes are present. Computer games with gay actors have not been criminalized yet, but competitions in these games are canceled. Some regions of Russia have announced gay-free. The Supreme Court of Russia decided that the use of feminitives is equal to participation in the “international LGBT movement [3].”

The TFR in Russia, as everywhere in the world, is declining. Now a very small cohort of Russian women is in the childbearing age. Therefore, the crude birth rate is at a historical nadir, in times of no significant progress in life expectancy. The Government deadly needed workers and soldiers and therefore exercise all imaginable and unimaginable means to turn history back. Unfortunately, all these efforts turn to evil.

Conflict of interest: None declared.
